# Association between dietary patterns and biological aging among Chinese middle-aged and older adults: evidence from a cross-sectional study

**DOI:** 10.3389/fnut.2026.1904010

**Published:** 2026-08-06

**Authors:** Xu Zhu, Luoyi Hu, Lina Guo, Shu Chen, Qiong Wu

**Affiliations:** 1 School of Integrated Chinese and Western Medicine, Hunan University of Chinese Medicine, Changsha, Hunan, China; 2 Medical School, Hunan University of Chinese Medicine, Changsha, Hunan, China; 3 School of Humanities and Management, Hunan University of Chinese Medicine, Changsha, Hunan, China

**Keywords:** biological aging, cross-sectional study, dietary patterns, KDM-BA acceleration, physiological dysregulation

## Abstract

**Background:**

Previous studies have shown that dietary patterns are associated with health outcomes in Chinese middle-aged and older adults. However, whether dietary patterns are associated with Klemera-Doubal biological age (KDM-BA) and physiological dysregulation (PD) remains unclear.

**Methods:**

This cross-sectional study included 4,846 adults aged 45 years and older from the 2009 wave of the China Health and Nutrition Survey (CHNS). Three dietary patterns were derived using principal component analysis based on energy-adjusted food group intakes (g/1,000 kcal): Southern pattern, Western-like pattern, and Plant-rich pattern. KDM-BA acceleration and PD were calculated from routine clinical biomarkers. Linear regression models were used to examine the associations between dietary pattern scores and the two aging measures. Restricted cubic spline regression was applied to evaluate potential non-linear dose-response relationships.

**Results:**

The Southern and Western-like patterns were inversely associated with KDM-BA acceleration (β = −0.47, 95% CI: −0.70, −0.24; *P* < 0.001 for the Southern pattern; β = −0.50, 95% CI: −0.74, −0.26; *P* < 0.001 for the Western-like pattern), whereas the Plant-rich pattern showed a positive association (β = 0.23, 95% CI: 0.01, 0.46; *P* = 0.043). For PD, only the Southern pattern was positively associated (β = 0.02, 95% CI: 0.01, 0.03; *P* < 0.001). Restricted cubic spline analysis revealed a non-linear relationship between the Western-like pattern and KDM-BA acceleration (*P* for non-linearity = 0.027), whereas the other two patterns were approximately linear.

**Conclusions:**

Dietary patterns were differentially associated with KDM-BA acceleration and PD. These findings suggest that specific dietary patterns are associated with healthy aging in Chinese middle-aged and older adults.

## Introduction

As population aging accelerates, delaying biological aging and extending healthspan have become key public health priorities worldwide. Conventional aging assessments based solely on chronological age fail to capture the interindividual heterogeneity in physiological decline. In recent years, researchers have developed various biological age metrics based on combinations of biomarkers. Among these, Klemera-Doubal biological age (KDM-BA) and the physiological dysregulation index (PD)—the latter calculated using Mahalanobis distance—have been shown to independently predict all-cause mortality and incident age-related diseases (1–5), offering more physiologically grounded and comprehensively integrative measures for aging research.

Diet is a key modifiable factor influencing biological aging. Unhealthy dietary patterns may accelerate multisystem physiological dysregulation and functional decline through chronic inflammation, oxidative stress, and insulin resistance (6). Studies have shown that adherence to the Mediterranean diet is associated with longer leukocyte telomere length (7), whereas high consumption of ultra-processed foods is linked to accelerated telomere shortening (8). Previous studies have shown that dietary patterns are associated with chronic disease and mortality in Chinese middle-aged and older populations (9, 10). However, it remains unclear whether dietary patterns are associated with composite aging indicators such as KDM-BA and PD. Given that individuals aged 45 years and older experience accelerated biological aging and are more susceptible to diet-related chronic diseases, we used data from the China Health and Nutrition Survey (CHNS) to examine the associations between dietary patterns and KDM-BA acceleration and PD in Chinese middle-aged and older adults.

## Materials and methods

### Study population

Data for this study were obtained from the 2009 wave of the China Health and Nutrition Survey (CHNS). The CHNS is an ongoing prospective cohort study that employs multi-stage random cluster sampling in nine provinces across China (Liaoning, Jiangsu, Shandong, Henan, Hubei, Hunan, Guangxi, Guizhou, and Heilongjiang). The aim of the CHNS was to examine the impact of socioeconomic and demographic changes, as well as dietary patterns, on the nutrition and health status of residents. The CHNS study protocol was approved by the institutional review boards at the University of North Carolina at Chapel Hill and the Institute of Nutrition and Health at the Chinese Center for Disease Control and Prevention, and all participants signed informed consent. After excluding participants aged <45 years (*n* = 5,869), those with missing outcome data (*n* = 1,252), those with missing dietary data (*n* = 50), and those with missing covariate data (*n* = 161), a final sample of 4,846 participants was included in the analysis. Compared with participants excluded due to missing data, those included in the final analysis were generally comparable in terms of demographic, lifestyle, and clinical characteristics (Supplementary Table S1). This suggests that the analytic sample was representative of the target middle-aged and older population.

### Measurement of biological age

Two measures of biological age were used in this study: Klemera-Doubal biological age (KDM-BA) and physiological dysregulation (PD). Both measures were calculated based on 12 routine clinical biomarkers: total cholesterol, triglycerides (natural logarithm), glycosylated hemoglobin, urea, creatinine, albumin, high-sensitivity C-reactive protein (natural logarithm), red blood cell count, platelet count, ferritin (natural logarithm), transferrin, and systolic blood pressure. These biomarkers cover multiple physiological dimensions, including cardiometabolic function, renal function, liver function, inflammation and immunity, and hematological parameters, which are consistent with those used in the study by Liu (5).

KDM-BA was calculated using the standard Klemera-Doubal algorithm, which estimates biological age as a weighted function of multiple biomarkers and chronological age (5). KDM-BA acceleration (KDM-BAacc) was then defined as the residual from regressing KDM-BA on chronological age. A positive KDM-BAacc indicates that biological age exceeds chronological age, reflecting accelerated aging, whereas a negative value indicates decelerated aging. PD was defined as the natural log-transformed Mahalanobis distance, which measures the degree of deviation of an individual's biomarker profile from a healthy reference population. Following the approach established in previous studies (5), we defined the reference population as participants aged 20–39 years with complete biomarker data, representing a period of minimal physiological dysregulation. A larger PD value indicates greater deviation from the healthy physiological state.

### Measurement of dietary intake

Dietary data were obtained from the three-day 24 h dietary recall survey conducted in the 2009 wave of CHNS. According to the Chinese food composition table, foods were classified into 19 groups: rice, wheat, other cereals, tubers, legumes, fungi and algae, vegetables, fruits, pork, other livestock meat, poultry, organ meat, aquatic products, milk, eggs, nuts, cakes, fast foods, and soft drinks (11). Average daily intake (g/d) and total energy intake (kcal/d) were calculated for each food group. To account for individual differences in total energy intake, energy-adjusted food intake was then calculated as grams of food consumed per 1,000 kcal of total energy intake (g/1,000 kcal). This energy-adjustment approach reflects dietary composition rather than absolute intake levels and has been widely used in dietary pattern studies.

### Covariates

Covariates were selected based on previous CHNS literature and their potential associations with biological aging (5). Demographic variables included age (continuous), sex (male or female), education level (categorized as middle school or below, high school, and college or above), place of residence (urban or rural), and annual household income per capita (categorized into tertiles: low, middle, and high). Lifestyle variables included sleep duration (short sleep <7 h, normal sleep 7–8 h, and long sleep >8 h), smoking status (current, former, or never), alcohol consumption (yes or no), body mass index, and total energy intake. Chronic conditions included hypertension, diabetes, myocardial infarction, and stroke. Physical activity was not included in the main models due to substantial missing data (53.6%) but was adjusted for in a sensitivity analysis. In addition, we conducted a separate sensitivity analysis further adjusting for antihypertensive and hypoglycemic medication use. We anticipated that unfavorable levels of these covariates (e.g., older age, current smoking, higher BMI, and presence of chronic diseases) would be associated with accelerated biological aging.

### Statistical analysis

Principal component analysis (PCA) was used to extract dietary patterns, with energy-adjusted food group intakes (g/1,000 kcal) as input variables. The Kaiser-Meyer-Olkin (KMO) measure was 0.54, and Bartlett's test of sphericity was significant (*P* < 0.001), suggesting that the correlation structure among food groups was acceptable for PCA. The number of principal components to retain was determined based on parallel analysis, the elbow of the scree plot, and the interpretability of the factor loadings.

Multiple linear regression models were used to analyze the association of dietary pattern principal component scores with KDM-BA acceleration and PD. Two models were constructed: Model 1 adjusted for age, sex, education, residence, sleep duration, smoking, alcohol, BMI, income, and total energy intake; Model 2 was further adjusted for hypertension, diabetes, myocardial infarction, and stroke. A sensitivity analysis adjusting for physical activity was performed in a subsample with complete PA data (Supplementary Table S3), due to substantial missing data for this variable. Linear regression assumptions were assessed for the fully adjusted model by inspecting the residual plot, Q-Q plot, and variance inflation factors. No serious violations of linearity or homoscedasticity were detected from the diagnostic plots (Supplementary Figure S1), and all VIF values were below 2.

Subgroup analyses were conducted by stratifying age ( ≤ 60 vs. > 60 years), sex, hypertension, and diabetes status, and interaction terms were added to the models to test for effect modification.

Restricted cubic spline regression was used to further examine the dose-response relationship between dietary pattern scores and KDM-BA acceleration. A likelihood ratio test was performed to assess non-linearity (12).

All statistical analyses were performed using R software version 4.1.2. A two-sided *P* value <0.05 was considered statistically significant.

## Results

A total of 4,846 Chinese adults aged 45 years or older were included in the analysis. The mean age was 59.29 years, and 52.95% of the participants were female. The majority had attained middle school or below (54.23%) and lived in rural areas (65.97%). A total of 63.70% of the participants reported normal sleep duration. Current smokers accounted for 28.37%, and 31.55% reported alcohol consumption. Other covariates were summarized in Table 1.

**Table 1:** Characteristics of the study participants.

Variables	All participants (*n* = 4,846)
Age, Mean ± SD	59.29 ± 9.94
BMI, Mean ± SD	23.48 ± 3.19
Total energy intake (kcal), Mean ± SD	1,653.11 ± 597.03
Sex, *n* (%)	
Male	2,280 (47.05)
Female	2,566 (52.95)
Education, *n* (%)	
Middle school or below	2,628 (54.23)
High school	2,057 (42.45)
College or above	161 (3.32)
Residence, *n* (%)	
Urban	1,649 (34.03)
Rural	3,197 (65.97)
Sleeptime, *n* (%)	
Long sleep	1,085 (22.39)
Normal sleep	3,087 (63.70)
Short sleep	674 (13.91)
Smoking, *n* (%)	
Former	205 (4.23)
Never	3,266 (67.40)
Now	1,375 (28.37)
Alcohol, *n* (%)	
No	3,317 (68.45)
Yes	1,529 (31.55)
Income, *n* (%)	
Low	1,511 (31.18)
Medium	1,544 (31.86)
High	1,791 (36.96)
Hypertension, *n* (%)	
No	3,915 (80.79)
Yes	931 (19.21)
Diabetes, *n* (%)	
No	4,634 (95.63)
Yes	212 (4.37)
Myocardial, *n* (%)	
No	4,776 (98.56)
Yes	70 (1.44)
Stroke, *n* (%)	
No	4,747 (97.96)
Yes	99 (2.04)

### Principal component analysis of dietary patterns

**Figure 1:**
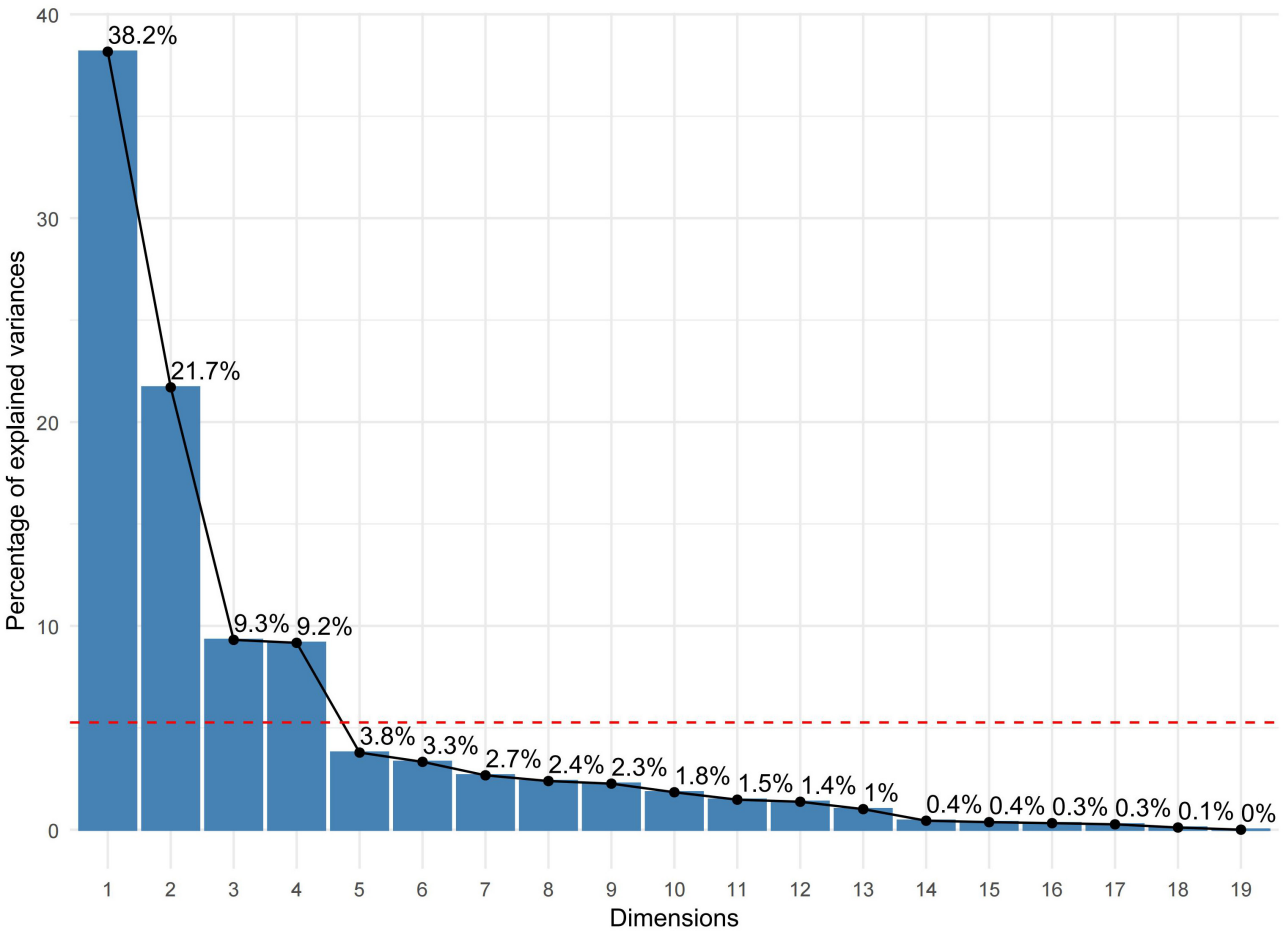
Scree plot of principal component analysis based on 19 food groups. The horizontal dashed line indicates an eigenvalue of 1. The first three components showed a clear inflection point and were retained for subsequent analyses.

Parallel analysis suggested five components, whereas the scree plot showed a clear inflection point at the third component (Figure 1). These three components yielded interpretable dietary patterns and were therefore retained for subsequent analyses. Together, they explained 38.17%, 21.70%, and 9.31% of the total variance, respectively, cumulatively accounting for 69.18% of the variance in food intake. The eigenvalues of the first three components were all greater than 1.

Table 2 presents the factor loadings for the three dietary patterns identified after varimax rotation. The first component, labeled as the Southern pattern, was characterized by high loadings for rice (0.81), pork (0.47), aquatic products (0.36), Organ meat (0.29), and poultry (0.27). This pattern reflects a dietary structure typical of southern China, where rice is the predominant staple food.

**Table 2:** Factor loadings for dietary patterns after varimax rotation.

Food group	Southern pattern	Western-like pattern	Plant-rich pattern
Rice	0.81	−0.13	0.14
Pork	0.47	0.06	−0.38
Wheat	−0.82	0.00	0.00
Poultry	0.27	0.21	−0.13
Aquatic products	0.36	0.38	0.07
Other cereals	−0.26	−0.07	−0.03
Tubers	0.13	−0.26	0.58
Eggs	−0.13	0.50	0.38
Milk	−0.03	0.58	−0.07
Fungi and algae	0.12	0.44	−0.07
Other meat	0.11	0.30	−0.04
Vegetables	0.19	0.03	−0.08
Cakes	−0.16	0.32	0.11
Nuts	−0.03	0.03	0.00
Organ meat	0.29	−0.06	−0.15
Legumes	0.02	−0.03	0.60
Soft drinks	0.00	−0.02	0.01
Fast foods	−0.14	0.15	−0.13
Fruits	0.00	0.27	0.49

The second component, labeled as the Western-like pattern, was defined by positive loadings for milk (0.58), eggs (0.50), fungi and algae (0.44), aquatic products (0.38), cakes (0.32), other meat (0.30), and fruits (0.27). This pattern represents a diverse dietary profile with high intakes of both animal and plant protein sources.

The third component, labeled as the Plant-rich pattern, was characterized by positive loadings for legumes (0.60), tubers (0.58), fruits (0.49), and eggs (0.38). This pattern suggests a plant-forward dietary preference with limited meat consumption.

### Dietary patterns and biological aging measures

For KDM-BA acceleration, each one-unit increase in the Southern pattern score was associated with a 0.47-year decrease (95% CI: −0.70, −0.24; *P* < 0.001). A similar association was observed for the Western-like pattern (β = −0.50; 95% CI: −0.74, −0.26; *P* < 0.001). In contrast, the Plant-rich pattern showed a positive association with KDM-BA acceleration (β = 0.23; 95% CI: 0.01, 0.46; *P* = 0.043).

For PD, the Southern pattern was positively associated with the outcome (β = 0.02; 95% CI: 0.01, 0.03; *P* < 0.001), whereas no significant associations were observed for the Western-like or Plant-rich patterns (Table 3). In a sensitivity analysis using the untransformed Mahalanobis distance, the results remained consistent (Supplementary Table S2).

**Table 3:** Associations between dietary patterns and biological aging measures.

Variable	Model 1 β (95% CI), *P*	Model 2 β (95% CI), *P*
KDM-BA acceleration		
Southern pattern	−0.45 (−0.68, −0.21), *P* < 0.001	−0.47 (−0.7, −0.24), *P* < 0.001
Western-like pattern	−0.62 (−0.87, −0.36), *P* < 0.001	−0.50 (−0.74, −0.26), *P* < 0.001
Plant-rich pattern	0.19 (−0.04, 0.43), *P* = 0.107	0.23 (0.01, 0.46), *P* = 0.043
PD		
Southern pattern	0.02 (0.01, 0.03), *P* < 0.001	0.02 (0.01, 0.03), *P* < 0.001
Western-like pattern	0.01 (<0.01, 0.01), *P* = 0.246	<0.01 (−0.01, 0.01), *P* = 0.777
Plant-rich pattern	−0.01 (−0.01, <0.01), *P* = 0.110	−0.01 (−0.02, <0.01), *P* = 0.061

Model 1 adjusted for age, sex, education, place of residence, sleep duration, smoking, alcohol consumption, BMI, income, and total energy intake. Model 2 further adjusted for hypertension, diabetes, myocardial infarction, and stroke.

Sensitivity analyses additionally adjusting for medication use and physical activity yielded results consistent with the main findings (Supplementary Table S3).

### Subgroup analysis

Significant interactions with sex were observed for the Southern pattern in relation to KDM-BA acceleration (*P* for interaction <0.001), with a stronger protective association in females (β = −0.84) than in males (β = −0.03) (Table 4). This interaction remained significant after Bonferroni correction (adjusted α = 0.004).

**Table 4:** Subgroup analyses for the association between dietary patterns and biological aging measures.

Subgroup	Southern pattern score β (95% CI)	Western-like pattern score β (95% CI)	Plant-rich pattern score β (95% CI)
KDM-BA acceleration			
Age			
≤ 60 years	−0.39 (−0.69, −0.09)	−0.45 (−0.79, −0.12)	0.21 (−0.10, 0.52)
> 60 years	−0.51 (−0.87, −0.16)	−0.57 (−0.93, −0.22)	0.26 (−0.08, 0.59)
*P* for interaction	0.425	0.846	0.792
Sex			
Male	−0.03 (−0.36, 0.30)	−0.37 (−0.74, 0.00)	0.46 (0.12, 0.80)
Female	−0.84 (−1.16, −0.53)	−0.56 (−0.88, −0.24)	0.06 (−0.24, 0.36)
*P* for interaction	<0.001	0.265	0.059
Hypertension			
No	−0.45 (−0.7, −0.2)	−0.51 (−0.78, −0.23)	0.36 (0.11, 0.61)
Yes	−0.54 (−1.11, 0.02)	−0.43 (−0.94, 0.09)	−0.28 (−0.78, 0.22)
*P* for interaction	0.378	0.458	0.045
Diabetes			
No	−0.48 (−0.71, −0.24)	−0.51 (−0.76, −0.27)	0.22 (−0.01, 0.45)
Yes	−0.42 (−1.88, 1.03)	−0.08 (−1.34, 1.19)	0.41 (−0.87, 1.69)
*P* for interaction	0.931	0.152	0.453
PD			
Age			
≤ 60 years	0.02 (0.01, 0.03)	0.00 (−0.02, 0.01)	−0.01 (−0.02, 0.00)
> 60 years	0.02 (0.01, 0.03)	0.00 (−0.01, 0.02)	−0.01 (−0.02, 0.00)
*P* for interaction	0.666	0.630	0.773
Sex			
Male	0.02 (0.01, 0.04)	0.00 (−0.01, 0.02)	−0.01 (−0.02, 0.00)
Female	0.02 (0.01, 0.03)	0.00 (−0.01, 0.01)	−0.01 (−0.02, 0.00)
*P* for interaction	0.362	0.775	0.432
Hypertension			
No	0.02 (0.01, 0.03)	0.00 (−0.01, 0.01)	−0.01 (−0.02, 0.00)
Yes	0.02 (0.00, 0.04)	0.01 (−0.01, 0.02)	0.01 (−0.01, 0.02)
*P* for interaction	0.567	0.496	0.077
Diabetes			
No	0.02 (0.01, 0.03)	0 (−0.01, 0.01)	−0.01 (−0.02, 0.00)
Yes	−0.02 (−0.08, 0.03)	0.01 (−0.04, 0.06)	0.00 (−0.05, 0.05)
*P* for interaction	0.035	0.781	0.786

### Dose-response relationships

**Figure 2:**
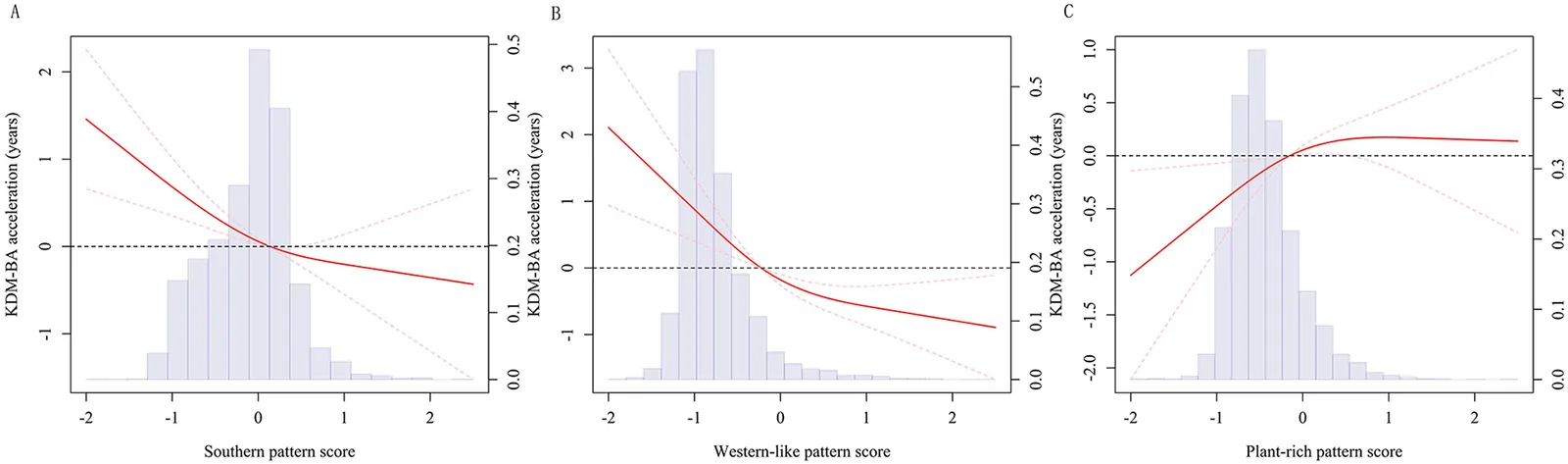
Non-linear dose-response relationships between dietary pattern scores and KDM-BA acceleration. **(A)** Southern pattern. **(B)** Western-like pattern. **(C)** Plant-rich pattern. Solid lines represent the estimated β coefficients, and dashed lines represent 95% confidence intervals. Histograms along the x-axis indicate the distribution of each dietary pattern score.

Restricted cubic spline analysis showed a significant overall association for all three dietary patterns with KDM-BA acceleration (overall *P* < 0.001 for the Southern pattern, overall *P* < 0.001 for the Western-like pattern, and overall *P* = 0.040 for the Plant-rich pattern). The Western-like pattern exhibited a non-linear relationship with KDM-BA acceleration (*P* for non-linearity = 0.027); the other two patterns were approximately linear (*P* for non-linearity = 0.160 for the Southern pattern and 0.127 for the Plant-rich pattern) (Figure 2). The RCS curve showed that the inverse association between the Western-like pattern and KDM-BA acceleration was strongest at lower to moderate pattern scores, with a gradual attenuation of the effect size at higher scores.

## Discussion

In this study, we used data from the 2009 CHNS to examine the associations between three dietary patterns and two biological aging measures in Chinese middle-aged and older adults. The Southern and Western-like patterns were inversely associated with KDM-BA acceleration, whereas the Plant-rich pattern showed a positive association. Only the Southern pattern was positively associated with PD. These findings contribute to the growing evidence linking dietary patterns to biological aging in Chinese populations, extending previous studies that have largely focused on Western populations or single aging indicators (13–16).

For KDM-BA acceleration, the Southern and Western-like patterns were both characterized by higher intakes of aquatic products, poultry, eggs, milk, and fungi and algae. These foods provide high-quality protein, n-3 polyunsaturated fatty acids, vitamin D, and B vitamins, which may delay biological aging through the inhibition of chronic inflammation and oxidative stress (17–19). Subgroup analyses showed that the inverse association between the Southern pattern and KDM-BA acceleration was more pronounced in women, which may be related to sex-specific metabolic regulation. Estrogen has been shown to modulate lipid metabolism, insulin sensitivity, and inflammatory responses, potentially enhancing the health benefits of dietary patterns rich in aquatic products and high-quality protein (20). In addition, sex differences in body composition and fat distribution may also influence the extent to which dietary components affect systemic inflammation and metabolic health. Compared with men, women tend to accumulate more subcutaneous adipose tissue and less visceral adipose tissue (VAT). From a metabolic perspective, subcutaneous fat is considerably less harmful than VAT, and this distribution pattern is generally associated with lower metabolic and cardiovascular risk (21).

The Plant-rich pattern was characterized by higher intakes of legumes, tubers, fruits, and eggs. Compared with the Southern and Western-like patterns, it had a more limited array of high-quality protein sources. As a result, this pattern may not provide adequate amounts of n-3 polyunsaturated fatty acids and B vitamins, both of which play important roles in suppressing chronic inflammation and maintaining normal metabolic function. This may partly explain why the Plant-rich pattern showed a positive association with KDM-BA acceleration.

For PD, the Southern pattern showed a significant positive association. This may be explained by the exceptionally high loading of refined white rice (0.81) in this pattern. White rice has a high glycemic index and its consumption has been linked to increased risk of type 2 diabetes, with each additional 150 g/day associated with a 6% greater risk (22). In Asian populations, higher rice consumption is associated with higher fasting glucose (0.81% per portion) and HOMA-IR (4.62% per portion), indicating greater insulin resistance (23). High-carbohydrate diets rich in refined grains are also positively associated with metabolic syndrome, particularly elevated blood pressure and triglycerides (24). These metabolic disturbances may increase an individual's Mahalanobis distance from the healthy reference profile, thereby elevating the PD score.

Unlike the Southern pattern, neither the Western-like nor the Plant-rich pattern showed a significant association with PD. The Western-like pattern, characterized by higher intakes of eggs, milk, aquatic products, and fungi and algae, had a nutrient profile that included both protective and potentially harmful components. On the one hand, n-3 polyunsaturated fatty acids from aquatic products exert anti-inflammatory effects, while milk and eggs provide high-quality protein and vitamin D (17–19). On the other hand, eggs and milk also supply saturated fat and animal protein, which may adversely affect lipid metabolism (25–28). These effects may offset each other across different physiological systems, resulting in no net effect on PD.

The Plant-rich pattern was characterized by higher intakes of legumes, tubers, fruits, and eggs. Legumes and fruits provide dietary fiber and antioxidants (29, 30), but the high glycemic index of tubers may induce metabolic fluctuations (31). In addition, this pattern has a limited range of high-quality protein sources, lacking the complementary contributions from aquatic products, poultry, and milk. The coexistence of protective and risk factors may result in a neutral net effect on multisystem physiological homeostasis.

The differential effects of the three dietary patterns on KDM-BA acceleration and PD underscore the multidimensional nature of biological aging assessment. KDM-BA acceleration captures the relative pace of aging, whereas PD reflects the level of physiological homeostasis (5). Thus, when evaluating the influence of diet on aging, it is important to consider both relative aging speed and multisystem physiological homeostasis.

Our findings have several implications for public health policy. The inverse association between the Southern pattern and KDM-BA acceleration suggests that maintaining traditional dietary habits—including higher intakes of rice, pork, aquatic products, organ meat, and poultry—may help delay biological aging in Chinese adults. However, the positive association between this pattern and PD highlights the need to reduce refined carbohydrate intake, particularly white rice, when promoting traditional diets. For clinical practice, these findings suggest that dietary counseling for middle-aged and older adults should consider both the quality and composition of protein and carbohydrate sources, rather than focusing solely on macronutrient quantity. Future longitudinal and intervention studies are needed to confirm causality and elucidate the biological pathways linking dietary patterns to different aging measures. Culturally tailored dietary guidelines informed by such evidence would facilitate healthy aging in China.

This study has several limitations. First, the cross-sectional design precludes causal inference. Second, the 3-day 24 h dietary recall may not accurately reflect long-term dietary habits. Third, despite adjustment for multiple covariates, residual confounding cannot be ruled out. Fourth, chronic conditions such as hypertension and diabetes may lie on the causal pathway between dietary patterns and biological aging; adjusting for these mediators may introduce overadjustment bias, and future longitudinal studies with formal mediation analyses are warranted to clarify the underlying pathways. Nevertheless, this study revealed the differential associations of dietary patterns with KDM-BA acceleration and PD in a large sample of Chinese middle-aged and older adults, and provides epidemiological evidence linking dietary patterns to biological aging.

## Data Availability

Publicly available datasets were analyzed in this study. This data can be found here: The data analyzed in this study are publicly available from the China Health and Nutrition Survey (CHNS) at https://chns.cpc.unc.edu/data/datasets/.
