# Supplementary material for: Association between dietary patterns and biological aging among Chinese middle-aged and older adults: evidence from a cross-sectional study
Source: Front Nutr. 2026 Aug 6;13:1904010. doi: 10.3389/fnut.2026.1904010 (PMC13492234; doi:10.3389/fnut.2026.1904010)
Supplement: Supplementary file 1 [file Supplementary_file_1.doc]

Supplementary Table S1. Comparison of baseline characteristics between included and excluded participants

|  |  | Excluded(n=1463) | Included(n=4846) | SMD |
| --- | --- | --- | --- | --- |
| Age (mean (SD)) |  | 60.21 (11.42) | 59.29 (9.94) | 0.09 |
| BMI(mean (SD)) |  | 23.34 (3.23) | 23.48 (3.19) | 0.04 |
| Total energy intake (kcal) (mean (SD)) |  | 1584.56  (586.67) | 1653.11 (597.03) | 0.12 |
| Sex (%) | Male | 728 (49.8) | 2280 (47.0) | 0.05 |
|  | Female | 735 (50.2) | 2566 (53.0) |  |
| Education (%) | Middle school or below | 720 (52.5) | 2628 (54.2) | 0.04 |
|  | High school | 600 (43.7) | 2057 (42.4) |  |
|  | College or above | 52 ( 3.8) | 161 ( 3.3) |  |
| Place (%) | Urban | 549 (39.8) | 1649 (34.0) | 0.12 |
|  | Rural | 832 (60.2) | 3197 (66.0) |  |
| Sleeptime (%) | Short sleep | 176 (12.9) | 674 (13.9) | 0.10 |
|  | Normal sleep | 823 (60.2) | 3087 (63.7) |  |
|  | Long sleep | 367 (26.9) | 1085 (22.4) |  |
| Smoking (%) | Never | 921 (66.7) | 3266 (67.4) | 0.05 |
|  | Former | 74 ( 5.4) | 205 ( 4.2) |  |
|  | Now | 386 (28.0) | 1375 (28.4) |  |
| Alcohol (%) | No | 922 (66.7) | 3317 (68.4) | 0.04 |
|  | Yes | 461 (33.3) | 1529 (31.6) |  |
| Income (%) | Low | 428 (32.4) | 1511 (31.2) | 0.05 |
|  | Medium | 392 (29.7) | 1544 (31.9) |  |
|  | High | 502 (38.0) | 1791 (37.0) |  |
| Hypertension (%) | No | 1108 (81.1) | 3915 (80.8) | 0.01 |
|  | Yes | 259 (18.9) | 931 (19.2) |  |
| Diabetes (%) | No | 1312 (95.9) | 4634 (95.6) | 0.01 |
|  | Yes | 56 ( 4.1) | 212 ( 4.4) |  |
| Myocardial (%) | No | 1354 (98.4) | 4776 (98.6) | 0.01 |
|  | Yes | 22 ( 1.6) | 70 ( 1.4) |  |
| Stroke (%) | No | 1349 (97.8) | 4747 (98.0) | 0.01 |
|  | Yes | 31 ( 2.2) | 99 ( 2.0) |  |

Supplementary Table S2. Sensitivity analysis comparing log-transformed PD and untransformed PD

| Variable | Log-transformed PD  β (95% CI), *P* | Untransformed PD  β (95% CI), *P* |
| --- | --- | --- |
| Southern pattern | 0.02 (0.01, 0.03), *P* < 0.001 | 0.07 (0.02, 0.12), *P* = 0.004 |
| Western-like pattern | 0.00 (-0.01, 0.01), *P* = 0.777 | 0.02 (-0.03, 0.07), *P* = 0.438 |
| Plant-rich pattern | -0.01 (-0.02, 0.00), *P* = 0.061 | -0.04 (-0.09, 0.01), *P* = 0.124 |

Supplementary Table S3. Sensitivity analysis additionally adjusted for medication use and physical activity

| Variable | Model 3  *β* (95% CI), P | Model 4  *β* (95% CI), P |
| --- | --- | --- |
| KDM-BA acceleration |  |  |
| Southern pattern | -0.47 (-0.71, -0.22),*P*<0.001 | -0.42 (-0.76,-0.09),*P*=0.013 |
| Western-like pattern | -0.52 (-0.78, -0.25),*P*<0.001 | -0.59 (-1.00,-0.18),*P*=0.004 |
| Plant-rich pattern | 0.24 (<0.01, 0.48),P=0.053 | 0.27 (-0.08,0.62),*P*=0.132 |
| PD |  |  |
| Southern pattern | 0.02 (0.01, 0.03),P<0.001 | 0.02 (0.01,0.03),*P*=0.002 |
| Western-like pattern | 0.00 (-0.01, 0.01),P=0.882 | 0.01 (-0.01,0.02),*P*=0.372 |
| Plant-rich pattern | -0.01 (-0.02, <0.01),P=0.053 | -0.01 (-0.02,<0.01),*P*=0.094 |

Model 3 adjusted for all covariates in Model 2 plus antihypertensive and hypoglycemic medication use.

Model 4 adjusted for all covariates in Model 2 plus physical activity.

Supplementary Figure S1. Diagnostic plots for the fully adjusted linear regression model
